# Research on the service quality of emergency medical language services during major unexpected public health events

**DOI:** 10.3389/fpubh.2023.1169222

**Published:** 2023-06-12

**Authors:** Jingyi Xie, Siyu Ma

**Affiliations:** ^1^International College of Chinese Studies, Fujian Normal University, Fuzhou, China; ^2^College of International Education, Capital Normal University, Beijing, China

**Keywords:** emergency medical language service, public health emergency, SERVQUAL, COVID-19, major unexpected public health events

## Abstract

**Introduction:**

Emergency Medical Language Services (EMLS) have played a crucial role in the COVID-19 pandemic. Research on the quality and its influencing factors of EMLS is necessary.

**Methods:**

This study used the SERvice QUALity (SERVQUAL) model to determine factors affecting the quality of EMLS during the pandemic. An online questionnaire was completed by 206 participants who received the service in 2021–2022. Structural Equation Modeling (SEM) indicated that the service provider and service process significantly influenced the Service Results.

**Results:**

In the service process, the evaluation of service content and responsiveness were highly correlated, and both factors significantly affected user satisfaction. In the service provider, tangibility and reliability were highly correlated. The key factors for user willingness to recommend the service were service content and tangibility.

**Discussion:**

Based on the results of the data analysis, it can be inferred that EMLS should be improved and upgraded in terms of service organization, talent cultivation, and service channel expansion. To enhance service organization, an emergency medical language team should establish a close collaboration with local medical institutions and government departments, and an EMLS center should be established with the support of hospitals, government, or civil organizations.

## 1. Introduction

EMLS are critical components of emergency services. These services are designed to provide emergency language assistance to individuals who require it during emergency situations. State and administrative agencies are responsible for offering EMLS to ensure that the public's fundamental rights are upheld in emergency situations. It includes services provided for different situations such as prevention and monitoring beforehand, response and disposal during emergencies, and intervention and consolation afterwards (1–3). In major public health emergencies, EMLS are an important tool for disaster relief practice. As early as after the 1995 Great Hanshin Earthquake, the Japanese academic community began researching and promoting the concept of “simple Japanese,” which was the predecessor of emergency language services. The emergency language services in the United States are primarily provided by the US Federal Emergency Management Agency, the US National Language Service Corps, and the US Emergency Medical Center's emergency medical language assistance program. These services mainly target individuals with limited English proficiency or hearing impairments within the United States, offering assistance during natural disasters, emergencies, and medical emergencies (4). These services provide references and inspirations for China's EMLS: EMLS should be used with foresight, organization, planning, and maturity, rather than being hastily responded to in a temporary and last-minute manner when encountering difficulties (5).

During 2021–2022, under the impact of the pandemic, the demand for EMLS for foreigners in China has surged. Medical institutions at all levels have collaborated with volunteer groups, schools, and others to launch related EMLS. These services mainly include emergency language translation, communication and the transmission of emergency medical information. Author and linguist Tony Thorne references the Chinese government who included linguists on the coronavirus outbreak team to explain medical terminology and health directives to patients (6). For example, the Shandong medical team that went to Hubei found a need for EMLS and created a concise language reference manual for emergency practice. In F city, during the epidemic, an EMLS team was formed by schools, hospitals, government departments, and volunteers. In the EMLS WeChat group for foreigners in China, the team provided medical information consultation, translation, and even bilingual accompaniment and instant translation communication services when necessary. The service was well-received by foreigners in F city, and the WeChat group reached its maximum capacity of 500 members. Some foreigners who were not in F city also joined the group to inquire about medical information. This article takes the individuals who receive EMLS as the research subjects, investigates their evaluations of the quality of EMLS, and identifies the various factors that affect service quality.

This kind of EMLS is a new development. Studies have shown that the current EMLS system, demand, and quality standards for public health crises are still in the exploratory stage. The exploratory nature of the survey questionnaire is helpful in identifying problems and finding factors that affect EMLS service quality. After the end of the epidemic, we also conducted follow-up interviews with those who were still in China, asking them how the services could be improved. This study examines the factors that influence the quality of EMLS for foreigners in China and discusses the various factors that affect EMLS evaluation to improve the efficiency of EMLS in the next public health crisis.

## 2. Related studies and theoretical research framework

This article discusses the factors that affect the quality of EMLS in public health institutions (such as hospitals) by analyzing the provider/ process/ result of EMLS in public health emergencies. Based on the emergency service quality survey model developed by Wang and Lin (7), this article refines the content of EMLS to improve service quality. The present study incorporates relevant literature on emergency language and medical services quality evaluation, quality management dimensions, and models. Specifically, prior research by Li and Shi (8), Miranda et al. (9), Tsu (10), Barrios et al. (11), and Ong et al. (12) is consulted to inform the theoretical framework and conceptualization of the study.

### 2.1. Research on EMLS and the quality of medical services

In previous studies [such as (7, 13)], the evaluation of EMLS quality can be divided into three parts: service provider, service process, and service result. The service provider includes individual competence, professional ability, and job practice. The service process includes service investment, service process, service content, and service format. The service result includes user experience and evaluation feedback. In our study, we mainly examined what factors affect the medical language service quality. Therefore, the study also takes into account the 4A standards of language services, namely Availability, Accessibility, Acceptability, and Adaptability (14). In conclusion, We regard the service provider and service process as the factors that affect the service result. This study discusses the factors that affect service quality, and on this model, we also need to define the response indicators of specific dimensions. Therefore, we introduced the SERVQUAL model.

### 2.2. Research on SERVQUAL dimensions

For many years, SERVQUAL has been used as a multidimensional scale with high validity and reliability and has been widely used to evaluate the quality of customer satisfaction and health-related organizations and other sectors, setting a benchmark for evaluating service quality among service providers. The five dimensions determined by Parasuraman et al. (15) have been used to evaluate the quality of public utility vehicle services in the Philippines (16). Similarly, German et al. (17) conducted an analysis on the factors that influence the preferences of Philippine consumers toward package carriers. In the healthcare sector, the SERVQUAL scale has been adapted to evaluate the quality of care and services perceived by patients in medical institutions (18, 19). Ocampo et al. (20) employed this framework to assess the quality of public services.

SERVQUAL scales typically consist of five parts: SERVQUAL scales typically comprise five dimensions: Reliability, Assurance, Tangibility, Empathy, and Responsiveness (16, 21). In healthcare quality evaluation, the adapted HEALTHcare service QUALity (HEALTHQUAL) scale typically includes four components: healthcare personnel, efficiency measures, non-healthcare personnel, and physical facilities (9), or five components: Empathy, Tangibles, Safety, Efficiency, and Improving Care Services (22). For instance, Lee and Kim's version (five dimensions and 32 items) and the HEALTHQUAL version adapted from Miranda et al. (9) previous work (four dimensions and 25 items) were utilized to examine service quality (9, 23).

On this basis, we have modified the scale for EMLS to better reflect the various factors that affect the quality of EMLS. Table 1 presents a comparative analysis between the proposed work and similar research studies, including state-of-the-art research.

**Table 1:** Comparison of EMLS research across literature.

**Research**	**Focus**	**Evaluation**	**Key contributions**
Li and Shi (8)	EMLS quality and medical services quality	Service provider, service process, and service result	Proposes a framework to evaluate the quality of EMLS and medical services
Miranda et al. (9)	Quality of care and services in medical institutions	Healthcare personnel, efficiency measures, non-healthcare personnel, and physical facilities	Provides a framework with HEALTHQUAL scale to assess the quality of healthcare services
Tsu (10)	Quality of medical translation and interpreting services	Quality assurance, quality control, and quality improvement	Proposes a framework to evaluate the quality of medical translation and interpreting services
Barrios et al. (11)	Quality of healthcare interpreter services	Service provider, service process, and service result	Identifies factors that affect the quality of healthcare interpreter services
Ong et al. (12)	Language barriers and healthcare communication	Linguistic and cultural competency, communication quality, and patient safety	Provides a framework to improve communication quality and patient safety in healthcare settings
Proposed work of this paper	Factors affecting the quality of EMLS in public health institutions during emergencies	Service provider, service process, and service results	By integrating Wang and Lin (7) emergency service quality survey model and the SERVQUAL model, the SERVQUAL-EMLS scale was developed, and it was used to measure the level of emergency service and its influencing factors in real-life situations in order to enhance service quality and identify factors that impact medical language service quality.

### 2.3. Theoretical research framework

This study aims to identify the factors that affect the quality of EMLS during major public health events. Therefore, the theoretical framework of this article is based on Wang and Lin (7) research on the Chinese emergency language service quality system, using the SERVQUAL model as the evaluation scale and combining the improvement of quality monitoring issues for medical staff in HEALTHQUAL. A new model has been developed.

In the model, since there are no non-service personnel in our emergency language service team, we have removed the option for non-medical personnel. In terms of service providers, the study focuses on the (institutional) reliability, (service channel) tangibility, and (service personnel) empathy of the service provider, corresponding to Wang and Lin (7) model of job practice, professional ability, and personal accomplishment. In terms of the service process, the input of service is not a factor that can be easily perceived by customers. Therefore, the present study excluded this item and utilized the dimensions of service content, responsiveness, and assurance to respectively measure the service provider, service process, and service form. At the same time, we use user satisfaction and service recommendation to evaluate service results, corresponding to “user experience” and “evaluation feedback.”

Compared with the SERVQUAL model, the model in this article mainly adds a “service content” dimension. Because the language content provided plays an important role in EMLS. Therefore, the researchers added an evaluation of the 4A standards for language content services (14) in the questionnaire. For example, “when the lockdown rules changed, I quickly received information” (Adaptability). “I received timely translation of the medical information I needed” (Accessibility and Availability), and so on.

The SERVQUAL standard model for EMLS (SERVQUAL-EMLS) includes three modules: service provider, service process, and service results. The service provider is measured by reliability, tangibility, and empathy. The service process is measured by service content, responsiveness, and assurance. The service results is measured by satisfaction and dissemination degree.

Based on this, 14 hypotheses were established in this article:

Firstly, the service results of EMLS are significantly affected by the service provider and service process, as demonstrated in the research by Wang and Lin (7), Chen (13), Liu and Yin (24), and others. Therefore, we hypothesize that:

(1) The service provider can positively influence the service results.
(2) The service process can positively influence the service results.

Secondly, the present study explored the relationship between the dimensions of reliability, tangibility, and empathy, which fall under the service provider, and the dimensions of service content, responsiveness, and assurance, which fall under the service process, and their impact on user satisfaction and service quality based on the SERVQUAL model. Previous studies have shown that reliability has a direct impact on user satisfaction and service quality. For instance, Shahin and Pourhamidi (25) revealed the association between service provider reliability and user satisfaction. Additionally, Zhao et al. (26) and Lee et al. (27) suggested that reliability influences user behavioral tendencies. Thus, it was hypothesized that:

(3) Reliability positively influences user satisfaction.
(4) Reliability positively influences dissemination degree.

According to several studies (16, 28–30), the relationship and emotions that exist between service providers and customers have a significant impact on both the dissemination degree and user satisfaction. Specifically, when service personnel demonstrate high levels of empathy, consumers tend to perceive their value as customers, which in turn positively affects the dissemination degree and satisfaction levels. Therefore, the variable of empathy represents one of the most crucial factors in fostering positive customer relations. Thus, it is hypothesized that:

(5) Service personnel empathy can have a positive impact on user satisfaction.
(6) Service personnel empathy can have a positive impact on dissemination degree.

Empirical evidence suggests that tangible equipment, machinery, and public facilities offered by service providers significantly influence user satisfaction (16, 31). In the context of EMLS, this is predominantly demonstrated through the provision of emergency medical vocabulary manuals, emergency medical guidance language manuals, emergency WeChat medical mutual aid platforms, and tangible language signs within hospitals. Thus, it is hypothesized that:

(7) Service tangibility can have a positive impact on user satisfaction.
(8) Service tangibility can have a positive impact on dissemination degree.

In EMLS, the accuracy of language and the richness of language types are also important factors that affect dissemination degree and satisfaction (7, 32). Therefore, it is hypothesized that:

(9) Service content can have a positive impact on user satisfaction.
(10) Service content can have a positive impact on dissemination degree.

Responsiveness is considered a crucial dimension in the service industry. Extant literature by Ocampo et al. (20), Lee et al. (27), and Chou et al. (33) has consistently suggested that the swiftness of response from service providers has a significant impact on both dissemination degree and customer satisfaction. Thus, responsiveness is deemed to have a highly positive and direct effect on both factors. In the context of EMLS, responsiveness is particularly critical (34). Thus, it is hypothesized that:

(11) Service responsiveness can have a positive impact on user satisfaction.
(12) Service responsiveness can have a positive impact on dissemination degree.

Assuring users of the quality and competency of the service is an effective approach adopted by the service industry to enhance dissemination degree and user satisfaction (30). Researchers such as Chuah and Hilmi (28) and Sam et al. (35) have highlighted the crucial role of this approach, particularly in the context of EMLS, where it is vital to instill a sense of professionalism and guarantee to users regarding the organization's dissemination degree (11, 36). Thus, it is hypothesized that:

(13) Assurance can have a positive impact on user satisfaction.
(14) Assurance can have a positive impact on dissemination degree.

**Figure 1:**
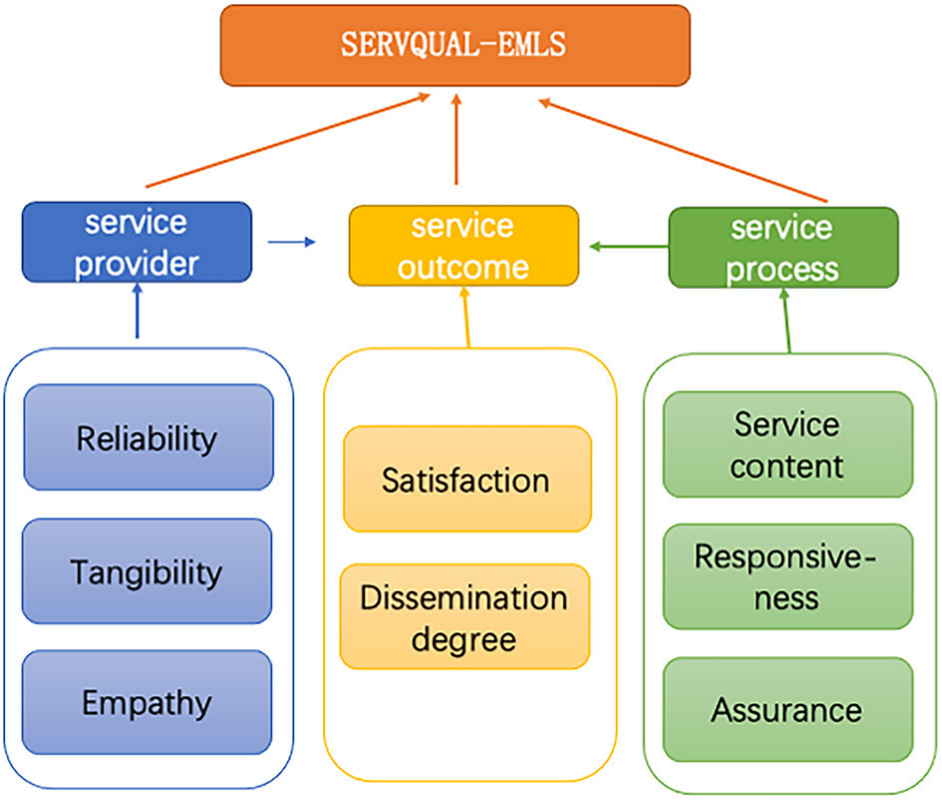
Initial research model.

In summary, the initial research model of this article is shown in Figure 1.

## 3. Methodology

### 3.1. Measurement

This study used a mixed-method approach, including both quantitative and qualitative analyses. In the first stage, five experts from service and academic fields in hospitals, government offices, and universities were invited to examine the questionnaire's validity. In the second stage, the quantitative method was used to collect data through the questionnaire survey. The questionnaire included items on service provider, service process, and service results, with sub-sections on reliability (3 items), tangibility (4 items), empathy (3 items), service content (3 items), responsiveness (4 items), assurance (3 items), satisfaction (3 items), and dissemination degree (3 items). Participants were asked to rate their level of agreement or disagreement with the statements on a 5-point Likert scale, ranging from 1 for “strongly disagree” to 5 for “strongly agree.” During the interview, we mainly conducted supplementary interviews to the corresponding population regarding the key points reflected in the questionnaire. All scale items in the questionnaire are listed in Table 2.

**Table 2:** Example of the scale section of the survey questionnaire.

Service provider	Reliability	Q59	We will regularly receive content push notifications for the service.
		Q56	During the epidemic, the service provided stable support for our medical needs.
		Q51	Whenever we ask a question, someone in the group will respond to our urgent medical needs.
	Tangibility	Q55	I can find the WeChat group for medical language services and also have the phone number of service personnel.
		Q49	In the hospital, I can find signs for EMLS.
		Q44	I regularly receive guides for medical services for foreigners in China.
	Empathy	Q27	I received a language handbook for medical services for foreigners.
		Q18	I believe that the service personnel really care about our health.
		Q17	I feel that the service personnel carefully consider our needs.
		Q16	When I was in the hospital, service personnel helped me communicate effectively with the doctor.
Service results	Satisfaction	Q54	I am quite satisfied with this medical language service.
		Q53	With the help of this information, I was able to get the medical services I needed.
		Q52	I think the messages I receive now are more accurate than those on FB and Twitter.
	Dissemination	Q40	If I encounter another emergency medical situation, I will choose this service again.
		Q33	I will recommend the hospital's official account, medical information groups, and mutual assistance groups to my friends.
		Q19	I think the coordination between the government, hospital, and community services is smooth.
Service process	Service content	Q57	I am satisfied with the health and medication knowledge provided by the service on the prevention and control of the new coronavirus.
		Q50	I am satisfied with the policy knowledge provided by the service on epidemic prevention and control, hospital visits, etc.
		Q45	The service provided me with the language I needed.
	Responsiveness	Q24	I received the medical information I wanted in a timely manner.
		Q23	When I encountered medical difficulties, I quickly received help.
		Q15	When communication with the doctor was not smooth, I could quickly get a translator.
		Q13	When the lockdown rules changed, I quickly received information.
	Assurance	Q46	I trust the information and service personnel in the group very much.
		Q41	I believe that the service complies with the legal regulations of the government, community, and hospital.
		Q14	Since the pandemic, I feel the assurance of their service during this pandemic.

### 3.2. Sample design and data collection

This study aimed to investigate the quality and satisfaction of EMLS received by foreigners living in City F during the COVID-19 pandemic. The survey used a mixed-methods design, combining both quantitative and qualitative data collection methods to gather a comprehensive understanding of participants' experiences and perceptions.

The sample for this study consisted of foreigners living in City F who had received EMLS at least once during the COVID-19 pandemic. The EMLS team provided offline services to some users, and for these individuals, they were asked to scan a Quick Response (QR) code and complete a paper questionnaire at the end of their service. For users requiring long-term services such as regular medical consultations, occasional medical advice and drug translation, and regular reception of epidemic prevention notices, the EMLS team sent online questionnaires *via* WeChat group from time to time to prompt them to fill out the questionnaire.

The survey methods for this study included on-site completion of paper questionnaires and mobile (QR code) questionnaires. A total of 206 valid questionnaires were collected. To reduce the potential biases associated with self-reported measures, follow-up visits were conducted with some of the foreigners who were still in China in January 2023 after the Chinese government announced the end of the pandemic. In addition to surveys, interviews and focus groups were also conducted to gather more in-depth and nuanced insights into participants' experiences and perceptions. This can help to triangulate the findings and increase the validity of the results.

The collected data was analyzed using SPSS26 and AMOS to provide an effective analysis of the data. The use of both quantitative and qualitative data collection methods allowed for a more thorough exploration of the research questions and helped to provide a more comprehensive understanding of the quality and satisfaction of EMLS received by foreigners living in City F during the COVID-19 pandemic.

### 3.3. Measurement model

In this study, we used Cronbach's Alpha coefficient to verify the reliability of the questionnaire, and Confirmatory Factor Analysis (CFA) to test the validity of the questionnaire's dimensions. Non-parametric analysis was used to analyze the effects of gender, age, education level, and Chinese proficiency on service results evaluation. Spearman correlation analysis was used to explore the relationships between each dimension and dissemination degree, satisfaction, and service results. SEM was used to discuss the relationships between each dimension, service quality, and satisfaction, as well as the overall relationship between all dimensions and service results.

## 4. Results

### 4.1. Descriptive statistics

Among the 206 samples collected in this study, 47.6% were male and 52.4% were female. The age distribution was mainly concentrated in the 18–25 and 26–30 age groups, accounting for 50.5% and 42.2% respectively, with relatively fewer participants aged over 30. The distribution of native languages was mainly English (57.3%), followed by French (26.7%), and other languages accounted for 15%. Most participants had a Chinese proficiency level of HSK Level 3 (39.8%), followed by HSK Level 4 (21.4%), and HSK Level 1 (18.4%). The data analysis results are shown in Table 3.

**Table 3:** Questionnaire sample distribution.

		**Frequency**	**Percentage%**
Gender	Male	98	47.6
	Female	108	52.4
Age	18–25	104	50.5
	26–30	87	42.2
	31–35	8	3.9
	>35	7	3.4
Native language	English	55	26.7
	French	118	57.3
	Others	33	16
Chinese language proficiency	HSK 1	38	18.4
	HSK 2	2	1
	HSK 3	82	39.8
	HSK 4	44	21.4
	HSK 5	19	9.2
	HSK 6	21	10.2

The data analysis results show that the service content and responsiveness during the service process were both close to 4 points, with scores of 3.82 and 3.63 respectively, indicating a relatively high level. However, assurance was close to 3 points with a score of 3.42, indicating a moderate level. The empathy and reliability of the service providers were both close to 3 points, with scores of 3.15 and 3.32 respectively, indicating a moderate level, while tangibility was close to 4 points, with a score of 3.55, indicating a slightly higher than average level. The satisfaction and dissemination degree of the service results were both close to 4 points, with scores of 3.54 and 3.52 respectively, indicating a relatively high level.

### 4.2. Analysis of differences

We analyzed the demographic characteristics of the respondents and found that gender, age, native language, and Chinese proficiency level all had an impact on their evaluation of the dimensions of the service.

The data analysis results show the gender has an impact on reliability, tangibility, satisfaction, and dissemination degree, and overall, females give higher ratings than males. Age has an impact on service content, responsiveness, reliability, tangibility, empathy, satisfaction, and dissemination degree, with older age groups giving lower ratings. Particularly for empathy and dissemination degree, in the age group of 30 and above, the average rating is already below 3 (Empathy ranges from 2.04 ± 1.27 in the 31–35 age group, and Dissemination degree ranges from 2.58 ± 0.97), and the majority of users in this age group are dissatisfied. The mother tongue of the service recipients has an impact on assurance, reliability, tangibility, and dissemination degree. The customers who are native speakers of English tend to provide the highest ratings, followed by those who are native speakers of French, whereas those whose mother tongue is other languages tend to provide comparatively lower ratings. The average rating for reliability, tangibility, and empathy among users whose mother tongue is a language other than Chinese is below 3, specifically 2.65 ± 0.97, 2.69 ± 0.96, and 2.85 ± 1.23, respectively. Additionally, language proficiency is found to have an impact on several service dimensions, namely service content, responsiveness, tangibility, empathy, and satisfaction. Specifically, higher proficiency in Chinese is associated with higher ratings for service content, whereas the ratings for reliability and tangibility tend to be lower among individuals with high proficiency in Chinese. In summary, individuals with higher proficiency in Chinese exhibit greater overall satisfaction levels.

Further interviews revealed that the older adults exhibits limited familiarity with the information dissemination methods employed by online platforms or WeChat groups, and consequently encounter difficulties in identifying appropriate social platforms or groups to access the requisite information. Such challenges can culminate in feelings of frustration, anxiety, and a sense of inadequacy. For this EMLS, they have a low level of trust in the information in WeChat groups, and the uptake rate of the distributed manuals and guide cards is also low. If their mother tongue is not English or French, they cannot understand neither Chinese or English on the EMLS manuals and need to use translation software, which reduces the quality of service and makes it difficult to obtain the approval of such users. Therefore, in EMLS, attention should be paid to the care of the older adults, especially older male adults, non-English-speaking mother tongue, and those with low proficiency in Chinese. Secondly, EMLS should strengthen communication with offline medical institutions and government agencies, which can increase service reliability and provide help in more practical application scenarios. Finally, EMLS should provide services in more languages within their capabilities, and provide more suitable and efficient translation tools or language assistants for users when resources are limited.

### 4.3. Reliability and validity

#### 4.3.1. Reliability

A total of 206 questionnaire data were collected in this survey. In order to verify the reliability of the questionnaire and whether the collected data could truly reflect the research intention, the reliability of the questionnaire needs to be verified first. According to the common standard, the Cronbach's Alpha coefficient is commonly used to measure the reliability of the survey. If the reliability coefficient is above 0.9, it indicates that the reliability of the questionnaire survey is good; if the reliability coefficient is >0.7, it is acceptable; if the coefficient is below 0.7, the questionnaire should be revised. The specific test results are shown in Table 4.

**Table 4:** The reliability of the survey questionnaire.

		**Cronbach's Alpha**	**Number of items**
Service process	Service content	0.821	3
	Responsiveness	0.845	4
	Assurance	0.875	3
	Overall	0.818	10
Service provider	Reliability	0.889	3
	Tangibility	0.905	4
	Empathy	0.905	3
	Overall	0.881	10
Service results	Satisfaction	0.910	3
	Dissemination degree	0.872	3
	Overall	0.809	6

The results from the table above show that the Cronbach's Alpha coefficients for all three scales and their dimensions are above 0.7, indicating a high level of reliability and good internal consistency for the scales used in this study. However, validated scales need to be further validated for their validity.

#### 4.3.2. Validity

**Figure 2:**
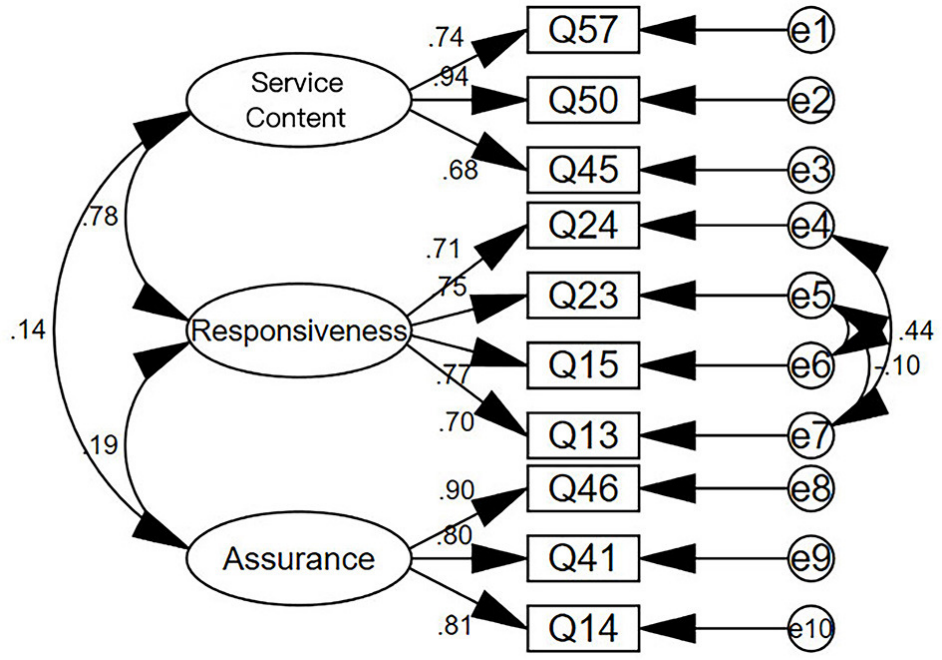
Structural equation model diagram of service process scale.

As the service process scale has been divided into three dimensions, the method of CFA was used to test its validity. A structural equation model diagram was established for the 206 valid questionnaires collected according to the three dimensions, and the following results were shown in Figure 2.

**Figure 3:**
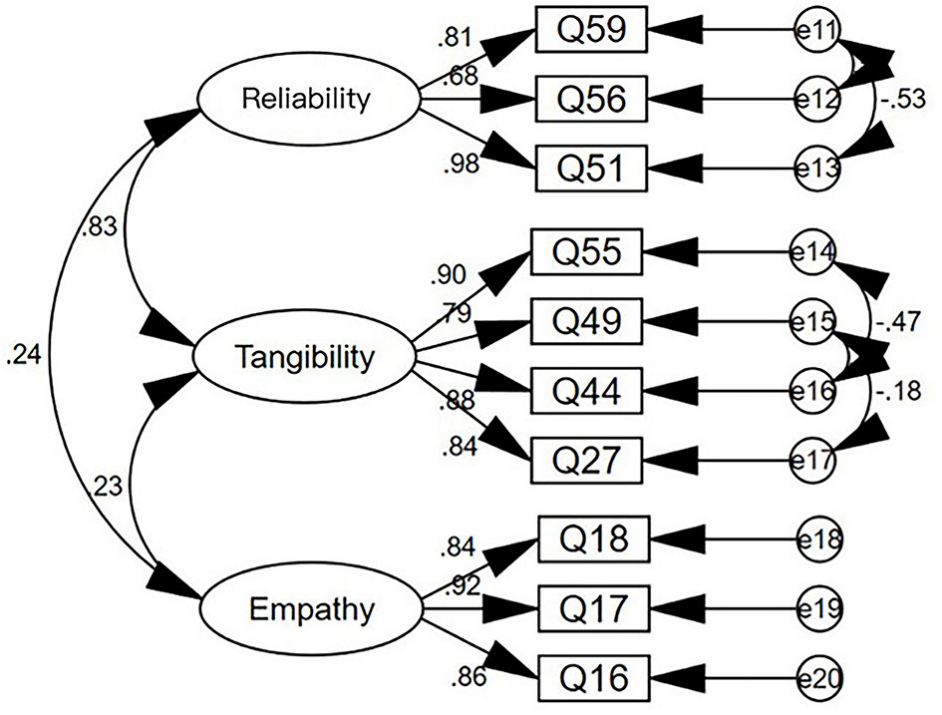
Structural equation model diagram of service provider scale.

As the service provider scale has been divided into three dimensions, the method of CFA was used to test its validity. A structural equation model diagram was established for the 206 valid questionnaires collected according to the three dimensions, and the following results were shown in Figure 3.

**Figure 4:**
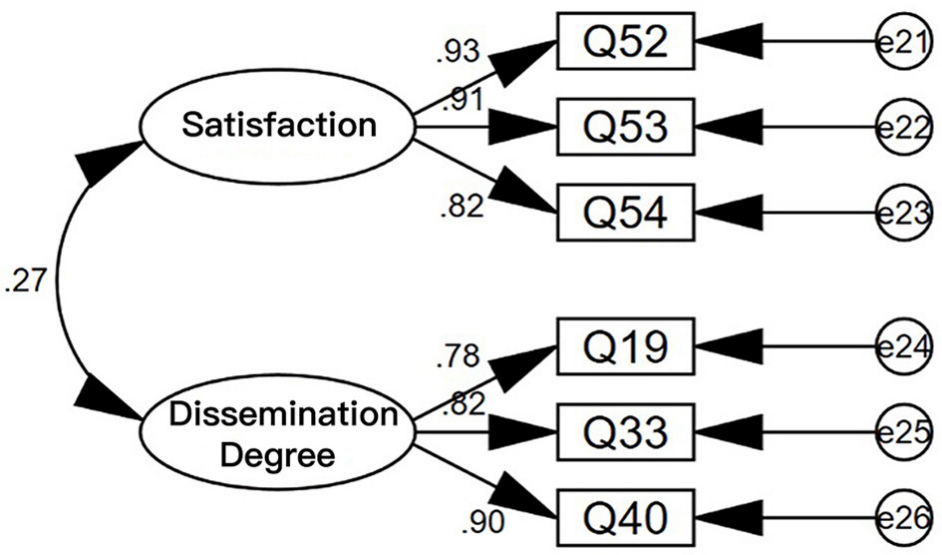
Structural equation model diagram of service result scale.

As the service result scale has been divided into two dimensions, the method of CFA was used to test its validity. A structural equation model diagram was established for the 206 valid questionnaires collected according to the two dimensions, and the following results are shown in Figure 4.

Table 5 shows the index values for the validation factor analysis model and the data of the service process, service provider, and service result scales.

**Table 5:** Index values for validating the factor analysis model of the scales.

**Fitting metric**	**CMIN/DF**	**RMR**	**RMSEA**	**GFI**	**AGFI**	**NFI**	**TLI**	**CFI**
Fitting criteria	< 5	< 0.05	< 0.08	>0.80	>0.80	>0.80	>0.80	>0.80
Computational output (service process scale)	2.109	0.066	0.074	0.942	0.891	0.945	0.954	0.970
Computational output (service provider scale)	4.107	0.061	0.123	0.906	0.809	0.934	0.914	0.948
Computational output (service result scale)	1.682	0.030	0.058	0.979	0.946	0.983	0.987	0.993

It can be seen from the data in the table that most of the indicators meet the requirements, indicating a good match between the model and the scale, and indicating that the model is valid.

Next, the convergence validity of the scale was analyzed. The main index for convergence validity is the Average Variance Extracted (AVE). The larger the AVE, the stronger the commonality of the measurement indicators, and the more they can reflect the same type of problem. The specific test results are shown in Table 6.

**Table 6:** Convergent validity analysis of the scales.

**Scales**	**Dimensions**	**Items**	**Factor loading**	**Reliability coefficient**	**Measurement error**	**CR**	**AVE**
Service process	Service content	Q57	0.74	0.54	0.46	0.83	0.63
		Q50	0.94	0.88	0.12		
		Q45	0.68	0.46	0.54		
	Responsiveness	Q24	0.71	0.50	0.50	0.82	0.54
		Q23	0.75	0.56	0.44		
		Q15	0.77	0.60	0.40		
		Q13	0.70	0.50	0.50		
	Assurance	Q46	0.91	0.82	0.18	0.88	0.70
		Q41	0.80	0.64	0.36		
		Q14	0.81	0.65	0.35		
Service provider	Reliability	Q59	0.81	0.66	0.34	0.87	0.69
		Q56	0.68	0.46	0.54		
		Q51	0.98	0.96	0.04		
	Tangibility	Q55	0.90	0.81	0.19	0.91	0.73
		Q49	0.79	0.62	0.38		
		Q44	0.88	0.77	0.23		
		Q27	0.84	0.71	0.29		
	Empathy	Q18	0.84	0.71	0.29	0.91	0.76
		Q17	0.92	0.84	0.16		
		Q16	0.86	0.73	0.27		
Service results	Satisfaction	Q52	0.93	0.86	0.14	0.87	0.69
		Q53	0.91	0.83	0.17		
		Q54	0.82	0.67	0.33		
	Dissemination degree	Q19	0.78	0.61	0.39	0.91	0.73
		Q33	0.82	0.67	0.33		
		Q40	0.90	0.82	0.18		

The factor loadings of the items in the service process, service provider, and service result scales are all above 0.5, indicating that all the items are valid.

The Composite Reliability (CR) of the dimensions of the service process, service provider, and service result scales are all above 0.6, and the AVE of the three dimensions are all above 0.5, indicating that the dimensions have good convergence validity.

In summary, it can be judged that the service process, service provider, and service result scales have good validity.

### 4.4. Bivariate correlations

Spearman correlation analysis is a commonly used tool to indicate the correlation between variables. In this article, we also use SPSS26 to conduct Spearman correlation analysis to show significant relationships between research variables. The results are presented in Table 7.

**Table 7:** Results of spearman correlation analysis.

	**Service content**	**Responsiveness**	**Assurance**	**Reliability**	**Tangibility**	**Empathy**	**Satisfaction**	**Dissemination degree**
Service content	1							
Responsiveness	0.637^**^	1						
Assurance	0.129	0.150^*^	1					
Reliability	0.326^**^	0.261^**^	−0.055	1				
Tangibility	0.145^*^	0.172^*^	0.164^*^	0.719^**^	1			
Empathy	0.139^*^	0.273^**^	0.180^**^	0.157^*^	0.200^**^	1		
Satisfaction	0.551^**^	0.497^**^	−0.004	0.292^**^	0.042	0.146^*^	1	
Dissemination degree	0.325^**^	0.278^**^	0.144^*^	0.311^**^	0.355^**^	0.259^**^	0.247^**^	1

* Correlation is significant at 0.05 level.

** Correlation is significant at 0.01 level.

From the table, it can be seen that service content, responsiveness, reliability, empathy are significantly positively correlated with Satisfaction, with all correlation coefficients >0 and *P* < 0.05. Service content, responsiveness, assurance, reliability, tangibility, empathy are significantly positively correlated with dissemination degree, with all correlation coefficients >0 and *P* < 0.05.

Among the above relationships, several groups of significant correlations have caught our attention:

In the service process, the evaluation of service content and responsiveness are highly correlated, and both significantly affect user satisfaction. The foregoing observation underscores the criticality of delivering prompt and efficacious information within the context of EMLS. In the course of our daily service provision, we have noted that users frequently seek translation and support for medical-related matters such as hospital regulations, physician's instructions, and medication usage, all of which necessitate swift and attentive responsiveness. Notably, even if the information conveyed is accurate and thorough, its efficacy diminishes once it exceeds the stipulated timeframe. Therefore, EMLS should have more manpower and resources to ensure timely response to user needs. During the lockdown period, this became particularly important. Many people choose EMLS because of urgent medical conditions. For example, a female student had an asthma attack during the epidemic but could not find appropriate medication, so she sought our help. We contacted some hospitals on her behalf and eventually found an alternative medication that satisfied her. However, on the other hand, our language service cannot replace medical care, and some people ask us for advice on which medication to take or which doctor to see, which our staff cannot answer. Going to find a doctor on behalf of the user actually prolonged the waiting time, thus reducing the user's satisfaction with the timeliness and content of our service.

The service provider is characterized by a strong correlation between tangibility and reliability. In our interviews, participants emphasized that even within social media platforms, the concept of tangibility remains crucial. The presence of tangible items such as a medical brochure or signage at physical hospital or community office locations imparts a sense of “reliability” and authenticity to the service, thus averting any potential for fraud or deception. Where tangible elements are absent, a stable phone number or designated medical service WeChat group can provide reassurance. In contrast, services confined solely to online platforms or applications tend to compromise their reliability, thereby contributing to lower user satisfaction with the quality of the service provided.

Whether users are willing to recommend your service to others, the content and tangibility of the service are more critical factors. In the era of the internet, online medical language information services have become one of the main service delivery methods. Content that can help others, such as providing knowledge about health care drugs for COVID-19, providing policy knowledge about epidemic prevention and hospital visits, having the language the other party needs, and so on, are more likely to be recommended by users. In the early stage of the epidemic outbreak, our medical service team also faced problems such as group inactivity, few users joining, and the supply and demand not matching. Later, the team introduced useful information collections such as “Collection of Major Hospital Visiting Guides” and “How to Make the Virus Go Away,” which successfully gained more recommended users to join the group and served more medical information demanders. If these pieces of information can be printed into physical flyers, concise and easy-to-use booklets that people can easily access in hospitals or communities, more readers can be obtained.

However, how these factors collectively affect service quality and their mutual impact model is analyzed through further SEM model analysis.

## 5. Confirmatory factor analysis and structural equation modeling

### 5.1. Path analysis from the SEM model of the dimensions

**Figure 5:**
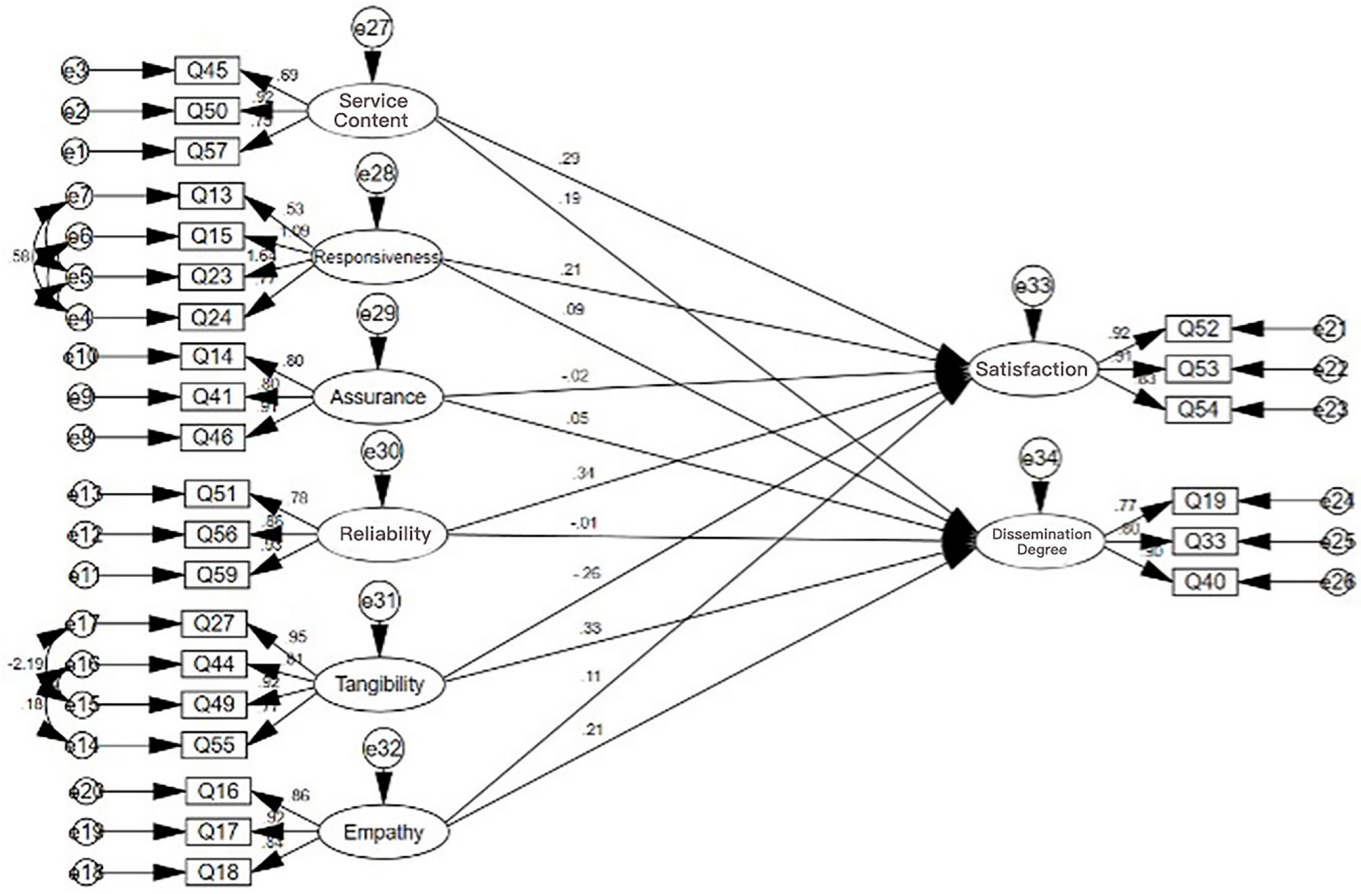
Path model of the influence of various dimensions of service provider and service process on service result dimensions.

To study the influence of the various dimensions of the service provider and service process on the service result dimension, the following path model is established as shown in Figure 5.

Based on the validation factor analysis model and scale data, it can be observed that the value of CMIN/DF is 3.854, which is less than the recommended threshold of 5. However, the value of CFI is 0.799, which is close to the standard level, and the other indicators are also relatively close to the standard. Despite the complexity of the model, the fit of the model to the actual data can be deemed generally acceptable. The SEM equation modeling table is presented in Table 8.

**Table 8:** Structural equation modeling table for each dimension.

			**Estimate**	**CR**	***P***
Satisfaction	< –	Service Content	0.293	4.525	^***^
Satisfaction	< –	Responsiveness	0.211	2.016	0.044
Satisfaction	< –	Assurance	−0.021	−0.341	0.733
Satisfaction	< –	Reliability	0.341	5.473	^***^
Satisfaction	< –	Tangibility	−0.262	−4.533	^***^
Satisfaction	< –	Empathy	0.111	1.825	0.068
Dissemination degree	< –	Service Content	0.185	2.544	0.011
Dissemination degree	< –	Responsiveness	0.086	1.508	0.132
Dissemination degree	< –	Reliability	−0.012	−0.174	0.862
Dissemination degree	< –	Tangibility	0.335	4.791	^***^
Dissemination degree	< –	Empathy	0.215	2.991	0.003
Dissemination degree	< –	Assurance	0.047	0.661	0.509

*** *p* < 0.001.

As can be seen from the table, in the satisfaction dimension, service content, responsiveness, and reliability can significantly influence satisfaction, with *P* < 0.05 and influence coefficients of 0.293, 0.211, and 0.341, respectively, all >0, indicating that service content, responsiveness, and reliability can all significantly positively influence satisfaction. For every 1 unit increase in service content, satisfaction will significantly increase by 0.293, 0.211, and 0.341. Tangibility can significantly negatively influence satisfaction, with *P* < 0.05 and an influence coefficient of −0.262 < 0, and for every 1 unit increase in tangibility, satisfaction will significantly decrease by 0.262.

In the dissemination degree dimension, service content, tangibility, and empathy can significantly positively influence dissemination degree, with *P* < 0.05 and influence coefficients of 0.185, 0.35, and 0.215, all >0. For every 1 unit increase in service content, tangibility, and empathy, dissemination degree will significantly increase by 0.185, 0.34, and 0.215, respectively.

This further illustrates the promotion relationship between service content, responsiveness, and reliability and user satisfaction. At the same time, if we want users to be more willing to recommend this service to others, we need to improve the quality of service content while also increasing tangibility and paying more attention to users' emotions.

Regarding the problem of how tangibility can increase the sharing rate but reduce user satisfaction, we found in user interviews that although tangible brochures are easy for users to distribute and give to neighbors, the content of the brochures is often not updated, the brochures are often given out, and there are too many and too messy brochures, which sometimes have a negative effect on user satisfaction. For example, one interviewee stated that they joined our WeChat group at the beginning of the pandemic and received the “Guide to Visiting Major Hospitals in F City.” However, when they got sick 1 day and arrived at the hospital, they found that the hospital's rules had completely changed. This made them very frustrated and they gave our service a rating of 1 point. Later, they also became a member of our EMLS team. It can be seen that the tangibility of EMLS does need to be improved, such as making the brochures lighter, publishing them more frequently, and printing the release time in a prominent position to remind people to check for the latest information, etc.

### 5.2. Path analysis from the SEM model of the whole

**Figure 6:**
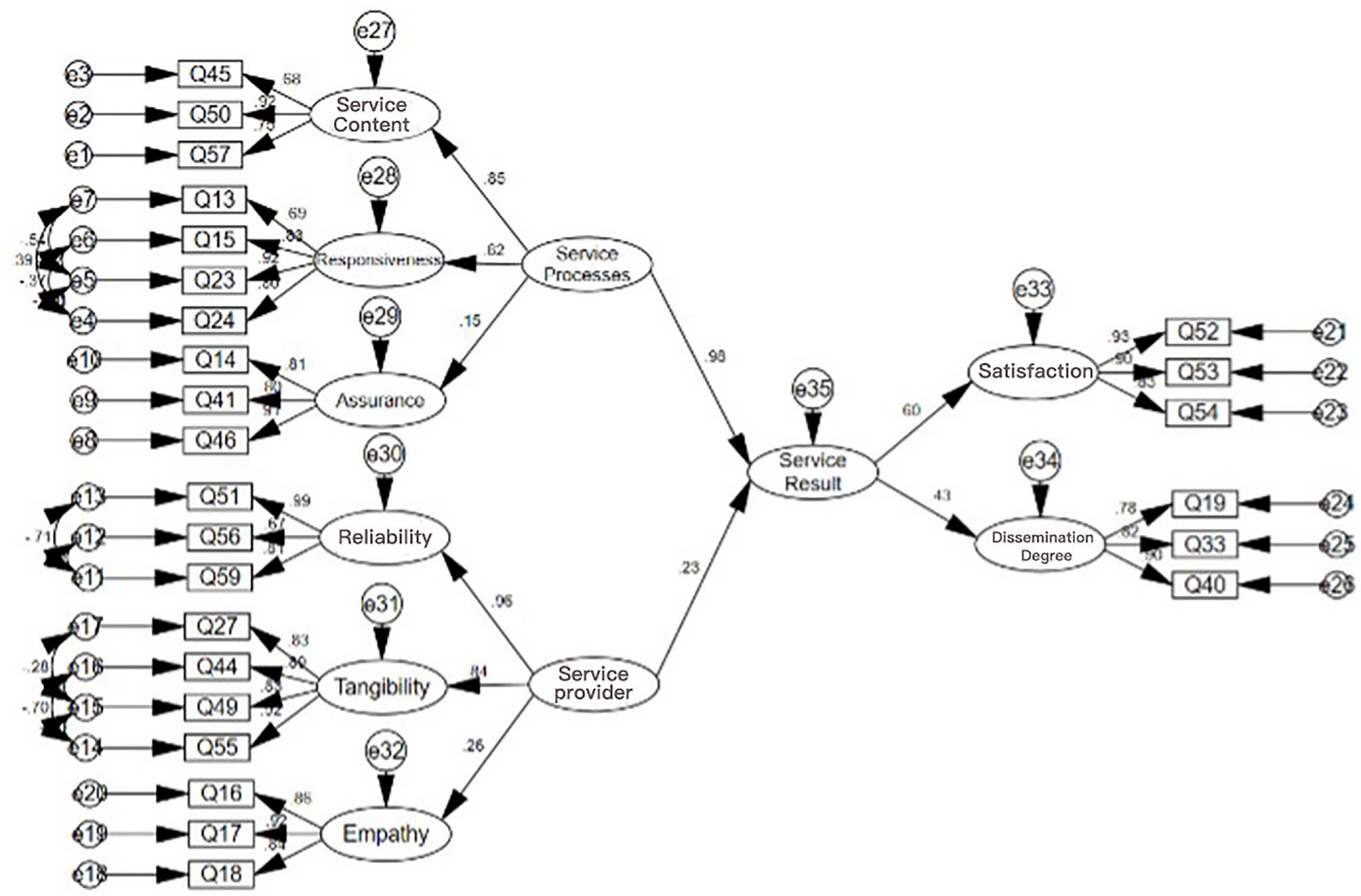
The overall influence of service providers and processes on service result dimensions.

To study the overall impact of the service provider and service process on the service result, the following path model was established: the overall impact of the service provider and service process on the service result dimension. The results are shown in Figure 6.

The index values of the verification factor analysis model and the scale data show that CMIN/DF is 2.845 < 5, while NFI = 0.816, TLI = 0.849, and CFI = 0.871 meet the standards. Most of the other indices are also close to the standards. Considering the complexity of the model and the real data, the fitting situation is generally acceptable. The overall SEM equation modeling table is shown in Table 9.

**Table 9:** Structural equation modeling table for the whole SEM.

			**Estimate**	**CR**	***P***
Service result	< –	Service process	0.984	6.743	^***^
Service result	< –	Service provider	0.226	2.292	0.022

*** *p* < 0.001.

From the table, it can be seen that:

The overall service process can significantly affect the overall service result, with *P* < 0.05 and an impact coefficient of 0.984 > 0. This means that the higher the overall service process, the more significant the service result. For every 1-point increase in the overall service process, the service result will significantly increase by 0.984.

The overall service provider can significantly affect the overall service result, with *P* < 0.05 and an impact coefficient of 0.226 > 0. This means that the higher the overall service provider, the more significant the service result. For every 1-point increase in the overall service provider, the service result will significantly increase by 0.226.

Therefore, it can be seen that both the service process and service provider can significantly improve the service result. Compared with the institutional construction of the service, users still place more emphasis on the service process. In EMLS, service personnel should focus on improving the quality of the service process to provide users with more accurate medical information, timely translation, information docking, and other services to optimize the service result and improve the overall service quality.

## 6. Discussion

### 6.1. Theoretical discussion

This study presents a framework for assessing service quality and identifying the factors that affect service outcomes in EMLS during a major public health crisis. The study integrates theories, SERVQUAL dimensions, and the EMLS Quality Framework to provide a unique perspective on measuring EMLS quality and user satisfaction during the COVID-19 pandemic. The study proposes a SERVQUAL-EMLS model to investigate the impact of each factor on service outcomes. Examining the comprehensive, integrated model can offer a deeper understanding of how to establish EMLS providers, optimize service processes, and implement measures to enhance service results, namely, improve user satisfaction and dissemination.

### 6.2. Practical implications

Based on the results of the data analysis, it can be inferred that EMLS should be improved and upgraded in terms of service organization, talent cultivation, and service channel expansion. To enhance service organization, an emergency medical language team should establish a close collaboration with local medical institutions and government departments, and an EMLS center should be established with the support of hospitals, government, or civil organizations.

The study found that the reliability of the service provider and satisfaction with the service content significantly impact service results. Given the uniqueness of medical information, EMLS should prioritize ensuring the professionalism and reliability of the information source to enhance the quality of service content. Such reliability should be established in daily cooperation mechanisms, with a focus on distributing critical information such as basic medical needs, hospital consultation rules, emergency rescue methods, and medical knowledge.

Talent cultivation is crucial, particularly in the areas of translation personnel and volunteer medical aid teams, to support the responsiveness, assurance, empathy, and other aspects of EMLS. The survey highlighted that people with poor language proficiency, especially those whose native language is not English, experienced significant difficulties seeking medical treatment or medication during the epidemic. Therefore, the development of medical glossaries, medical terminology collections, etc., specifically for EMLS, is essential to assist them in obtaining timely assistance from EMLS.

The implementation of the SERVQUAL-EMLS model is accompanied by costs such as staff training, interpreter hiring, and software acquisition, among others. However, the long-term benefits of enhancing patient satisfaction and reducing errors in care could surpass the costs incurred. The cost-effectiveness of the model is influenced by the healthcare organization's size, capacity, and patient population. In particular, organizations with a large volume of non-English speaking patients may find the SERVQUAL-EMLS model more economically viable, and thus promoting it in designated hospitals for foreigners seeking medical treatment is a more feasible approach.

Despite the potential benefits, several barriers to implementing the SERVQUAL-EMLS model exist. These include staff or patient resistance, inadequate translation services or volunteer resources, and the difficulty in customizing the model to suit the organization's requirements. Additionally, implementing an EMLS system may pose more significant challenges during non-crisis periods as healthcare organizations may not be willing to stockpile resources for emergencies. As such, successful implementation requires coordination and cooperation between various departments.

It is evident that the deployment of EMLS requires a comprehensive support system, including coordinated policies, laws, and technical applications, as well as the establishment of efficient and expedient information communication channels. For instance, an online multilingual medical information consultation module for foreigners could be added to the already mature e-government information release platform. The government, medical, hospital, insurance, and other systems should collaborate and communicate to enable emergency medical service teams to obtain relevant information more transparently and efficiently, thereby providing better service.

### 6.3. Limitations and future research

In addition to the theoretical and practical contributions, this study has several limitations. Firstly, the study was conducted in only one city in China, and it is recommended that future research should include other emergency medical language service providers from other regions to measure the quality and satisfaction of EMLS during a pandemic using the integrated framework. Secondly, the study was conducted during the COVID-19 pandemic, and the strict lockdown policies may have affected the sample size and representativeness. Therefore, future studies should consider the limitations of the sample, including potential lack of representativeness and possible biases associated with self-reported measures. Moreover, the perspectives of the EMLS service providers could be included to provide a more comprehensive understanding of the service quality.

Furthermore, as all service providers in this study were volunteers and did not receive any compensation, it is important to discuss the cost-effectiveness and feasibility of implementing the proposed SERVQUAL-EMLS model in practice, including potential barriers and facilitators. Future studies could investigate the impact of the pandemic and other major public health crises on EMLS in different regions and countries. Additionally, it would be valuable to obtain the views and improvement suggestions of healthcare professionals, nurses, and government health officials on EMLS services.

## 7. Conclusion

This study is the first to use a data model to analyze the quality of EMLS during the epidemic period. The analysis shows that the quality assessment of EMLS can be divided into three modules: service provider, service process, and service results. The service provider and service process will affect the service results. The user's gender, age, mother tongue, and Chinese proficiency may also affect their perception of service quality.

This study also explores the factors that affect the quality of EMLS during major public health emergencies, revealing the impact of service content, responsiveness, assurance, reliability, tangibles, and empathy on service satisfaction and dissemination. The results show that improving the institutional construction, talent training, and service channel of EMLS can effectively enhance the service quality of EMLS during major public health emergencies.

The research results will provide decision-making suggestions for hospitals, government agencies, and other entities that provide EMLS. It is hoped that our research can improve the emergency service level of medical institutions and government health departments, and provide better EMLS in the event of major public health emergencies in the future. It is also hoped that experiences can be shared with other EMLS providers, groups, and individuals to jointly prepare for possible crises.

## Data availability statement

The raw data supporting the conclusions of this article will be made available by the authors, without undue reservation.

## Ethics statement

The studies involving human participants were reviewed and approved by Fujian Normal University Ethics Committee. The participants provided their written informed consent to participate in this study.

## Author contributions

JX contributed to conceptualization, literature review, methodology, software, and writing. SM contributed to literature review and data collection. All authors contributed to the article and approved the submitted version.
